# A Fast and Accessible Method for the Isolation of RNA, DNA, and Protein To Facilitate the Detection of SARS-CoV-2

**DOI:** 10.1128/JCM.02403-20

**Published:** 2021-03-19

**Authors:** Jose Carlos Ponce-Rojas, Michael S. Costello, Duncan A. Proctor, Kenneth S. Kosik, Maxwell Z. Wilson, Carolina Arias, Diego Acosta-Alvear

**Affiliations:** a Department of Molecular, Cellular, and Developmental Biology, University of California, Santa Barbara, Santa Barbara, California, USA; b Neuroscience Research Institute, Santa Barbara, California, USA; c Center for BioEngineering, University of California, Santa Barbara, Santa Barbara, California, USA; St. Jude Children's Research Hospital

**Keywords:** field deployable, rapid isolation of RNA, DNA and protein, SARS-CoV-2, virus detection

## Abstract

Management of the coronavirus disease 2019 (COVID-19) pandemic requires widespread testing for severe acute respiratory syndrome coronavirus 2 (SARS-CoV-2). A main limitation for widespread SARS-CoV-2 testing is the global shortage of essential supplies, among them RNA extraction kits. The need for commercial RNA extraction kits places a bottleneck on tests that detect SARS-CoV-2 genetic material, including PCR-based reference tests. Here, we propose an alternative method we call PEARL (precipitation-enhanced analyte retrieval) that addresses this limitation. PEARL uses a lysis solution that disrupts cell membranes and viral envelopes while simultaneously providing conditions suitable for alcohol-based precipitation of RNA, DNA, and proteins. PEARL is a fast, low-cost, and simple method that uses common laboratory reagents and offers performance comparable to that of commercial RNA extraction kits. PEARL offers an alternative method to isolate host and pathogen nucleic acids and proteins to streamline the detection of DNA and RNA viruses, including SARS-CoV-2.

## Abstract (precis)

Management of the coronavirus disease 2019 (COVID-19) pandemic requires widespread testing for severe acute respiratory syndrome coronavirus 2 (SARS-CoV-2). A main limitation for widespread SARS-CoV-2 testing is the global shortage of essential supplies, among them RNA extraction kits.

## INTRODUCTION

The coronavirus disease 2019 (COVID-19) pandemic has had a devastating social and economic impact worldwide. As the disease continues to spread, the need for global severe acute respiratory syndrome coronavirus 2 (SARS-CoV-2) testing is more urgent than ever. The most widely used reference test for SARS-CoV-2 detection relies on the isolation of viral genetic material followed by PCR-based amplification (1, 2). The first step in this approach is the extraction of viral RNA from human samples. Commercial solid-phase RNA extraction kits that isolate viral RNA are the starting point for PCR-based SARS-CoV-2 reference tests (3). These kits use silica-based columns to purify viral RNA after disruption of cells and viral particles with proprietary reagents. The global demand for these kits has made them a limiting resource for SARS-CoV-2 testing, fueling the development of alternative SARS-CoV-2 RNA isolation methods and protocols. These alternative approaches include organic solvent-based RNA extraction and the use of chaotropic agents and proprietary buffer formulations.

TRIzol, a phenol- and guanidine-based reagent routinely used for isolation of RNA, DNA, and proteins, has been used to isolate SARS-CoV-2 RNA (4–6). However, TRIzol extraction is labor-intensive, which challenges scaling up to meet testing demands. Moreover, it requires special considerations for the disposal of organic solvents. A 5-min RNA preparation method was recently reported, but it depends on expensive proprietary lysis solutions originally developed for genomic DNA isolation (7). Recently, direct detection of SARS-CoV-2 in nasopharyngeal swab samples without RNA extraction was reported, indicating that the initial RNA isolation step could be omitted (8–10). Despite encouraging results, this approach results in reduced sensitivity of downstream quantitative PCR (qPCR)-based detection. On average, this method required an additional 5 to 7 PCR cycles to reach the detection threshold compared to that of reactions with purified RNA as the template. Because detection of low viral loads is critical for minimizing false-negative results, it is essential that new approaches not compromise sensitivity. In a more recent report, guanidium chloride was used for sample lysis in nasal swabs obtained from COVID-19-positive patients (11). Total RNA was subsequently precipitated with isopropanol. This approach conveniently concentrates the RNA, which can increase detection sensitivity in downstream analyses. However, the use of the toxic chaotropic agent guanidium chloride requires special disposal procedures.

To address the aforementioned shortcomings, we developed a simple technique to isolate nucleic acids and proteins from cells and viruses we call PEARL (precipitation-enhanced analyte retrieval). PEARL is fast, is easy to perform, and uses common laboratory reagents. Moreover, PEARL allows the downstream detection of specific SARS-CoV-2 viral sequences with sensitivity comparable to that afforded by commercial RNA extraction kits. PEARL can be coupled to nucleic acid amplification or immunodetection methods to detect host and viral RNA, DNA, and proteins from multiple sources. PEARL does not require specialized equipment or highly trained personnel, and it offers a low-cost straightforward alternative to facilitate virus detection.

## MATERIALS AND METHODS

### PEARL.

Samples were mixed in a 1:2 (vol/vol) sample-lysis solution [0.5% IGEPAL CA-630, 450 mM sodium acetate, 20% glycerol, 20 mM Tris(2-carboxyethyl)phosphine hydrochloride (TCEP), 50 μg/ml linear polyacrylamide, and 20 mM HEPES-KOH (pH 7.2)] ratio and incubated for 5 min at room temperature. Next, nucleic acids and proteins were precipitated on ice, for 10 min, using 1 volume of cold isopropanol. The precipitated material was collected by centrifugation at 19,000 × *g* for 10 min, washed once with 75% ethanol, air dried for 5 min at room temperature, and solubilized in 20 μl of nuclease-free water for amplification-based detection of nucleic acids or immunodetection of proteins. Analyses of the integrity of DNA, RNA, and protein were performed by nuclease/protease digestion and gel electrophoresis as follows: RNase A (Ambion), 5 μg/μl, 15 min at 37°C; DNase I (New England Biolabs), 0.2 U/μl, 30 min at 37°C; proteinase K (Macherey-Nagel), 2 μg/μl, 30 min at 37°C. Total RNA was isolated from PEARL extracts using TRIzol (Thermo Fisher) following the manufacturer’s recommendations.

### Clinical specimens.

Residual nasopharyngeal, nasal, and oropharyngeal samples previously tested for SARS-CoV-2 were obtained from the Santa Barbara County Department of Public Health and the University of California San Francisco Clinical Laboratories at China Basin.

### Cell culture and infections.

All cell lines were maintained in Dulbecco's modified Eagle's medium (DMEM) supplemented with 10% fetal bovine serum (FBS), l-glutamine, and antibiotics (penicillin/streptomycin, 100 U/ml) and were maintained in a humidified incubator at 37°C and 5% CO_2_. iSLK-219 cells are latently infected with KSHV.219 (12). This recombinant virus is maintained in cells as an episome. Green fluorescent protein (GFP) is constitutively expressed from the episome under the control of the human EF1 promoter. iSLK-219 cells also harbor the gene for a doxycycline-inducible KSHV replication and transcription activator (RTA). Uninfected iSLK and iSLK-219 cells were grown to 80% confluence, collected by trypsinization after two washes with phosphate-buffered saline (PBS) (GenClone), counted, and diluted at the desired density in 250 μl of PBS for PEARL extraction. For Zika virus (ZIKV) infections, HeLa cells were grown to 60% confluence and then infected with ZIKV at a multiplicity of infection (MOI) of 1. At 48 h postinfection, the cells were collected by trypsinization after two washes with PBS (GenClone) and counted. Cells were diluted in 250 μl of PBS for PEARL extraction.

### qPCR.

PEARL extracts were obtained from deidentified human samples or cultured cells. SARS-CoV-2-positive human samples were heat inactivated by incubation at 56°C for 30 min before RNA extraction. RNA from these samples was obtained with the QIAamp Mini Elute virus spin kit (Qiagen) following the manufacturer’s protocol, using 200 μl of sample input and eluting the purified RNA in 50 μl. PEARL extracts were prepared using 250 μl of SARS-CoV-2-positive human samples or a fixed number of cultured infected cells suspended in 250 μl of PBS. PEARL extracts from cultured cells were treated with either DNase I (1 U per 8 μl of PEARL extract; New England Biolabs) or with RNase A (0.1 mg per 8 μl of PEARL extract; Thermo Fisher) in a final volume of 10 μl for 30 min at 37°C. Five microliters of DNase-treated samples was reverse transcribed in a final volume of 10 μl using the iScript cDNA synthesis kit (Bio-Rad) following the manufacturer’s protocol and diluted 5-fold in nuclease-free water before qPCR. Target detection by qPCR was carried out with SYBR Select master mix (Applied Biosystems) using 2 μl of diluted cDNA as the template, and following the manufacturer’s protocol. The entire 10 μl from RNase-treated samples (genomic DNA) were diluted 5-fold with nuclease-free water, and 2 μl of diluted genomic DNA was used as the template for detection of specific genes with the SYBR Select master mix (Applied Biosystems) following the manufacturer’s protocol. Detection of SARS-CoV-2 N1 gene sequences and host RNase P mRNA from deidentified SARS-CoV-2-positive samples was carried out with the one-step TaqMan RNA-to-Ct 1-Step kit (Thermo Fisher), using 2 μl of undiluted PEARL extract and following the manufacturer’s protocol. All qPCR data were collected using a CFX96 Touch real-time PCR instrument (Bio-Rad) and analyzed with the CFX Maestro 1.1 software (Bio-Rad). Quantification cycle (*C_q_*) values were determined by regression. Data analysis and statistical tests were performed using Graph Pad Prism 6.0 software.

### Immunodetection.

Nuclease-treated PEARL extracts were separated on 10% SDS-PAGE gels and transferred onto nitrocellulose membranes (Bio-Rad) for Western blot analysis. The membranes were blocked in 0.5% bovine serum albumin–Tris-buffered saline with Tween (BSA-TBST) for 30 min. Primary antibodies were diluted in 0.5% BSA-TBST as follows: anti-HSP-70 (Cell Signaling Technology 4872), 1:1,000; anti-LANA/ORF73 (Advanced Biotechnologies 13–210-100), 1:3,000; anti-GFP (Invitrogen A11122), 1:3,000; and anti-NS2B (GeneTex GTX133308), 1:1,000. The membranes were incubated with primary antibodies for 30 min at room temperature. Following primary-antibody incubation, the membranes were washed with TBST 3 times before the addition of horseradish peroxidase (HRP)-conjugated secondary antibodies. The membranes were incubated for 30 min with secondary antibody diluted 1:3,000 in 0.5% BSA-TBST. Immunoreactivity was detected with Radiance Plus HRP substrate (Azure Biosystems). All images were captured with an Azure Biosystems C300 gel imaging system. Image postprocessing was carried out in Photoshop CC (Adobe) using automatic contrast. For dot blot-based immunodetection, nitrocellulose membranes (Bio-Rad) were spotted with 1 μl of PEARL extract and allowed to dry completely at room temperature for 30 min. For the remainder of the procedure, membranes were processed and imaged as described for Western blotting.

### Hand-powered centrifuge.

Our hand-powered centrifuge was designed in SolidWorks 2018 (Dassault Systèmes), sliced (0.2-mm layer height) in Cura (Ultimaker), and printed on an Ender3 3D printer (Creality) using a 1.75-mm polylactic acid filament (Hatchbox Inc.). To actuate our device, we used Brutal Strong 135-test braided fishing line (Izorline International). Approximately 1 m of line was threaded through holes designed for the string-driven system in the hand pulls and in the rotor, and the line was secured to itself with a double uni knot forming a loop. Maximum angular speed was determined by affixing reflective tape to the rotor, and revolutions per minute were measured with a laser tachometer (Neiko) over a 1-s sampling time. The maximum relative centrifugal force (RCF) was calculated as 1.118 × 10^−5^ × *r*_max_ × rpm^2^, where *r*_max_ is specified in centimeters.

3D print files can be found at https://3dprint.nih.gov/discover/3dpx-014683. After PEARL precipitation, the samples were spun at maximum speed with the hand-powered centrifuge or at 19,000 × *g* in a benchtop centrifuge. RNA and protein recovery for both centrifugation methods was determined by RT-qPCR and dot blotting.

## RESULTS

**FIG 1:**
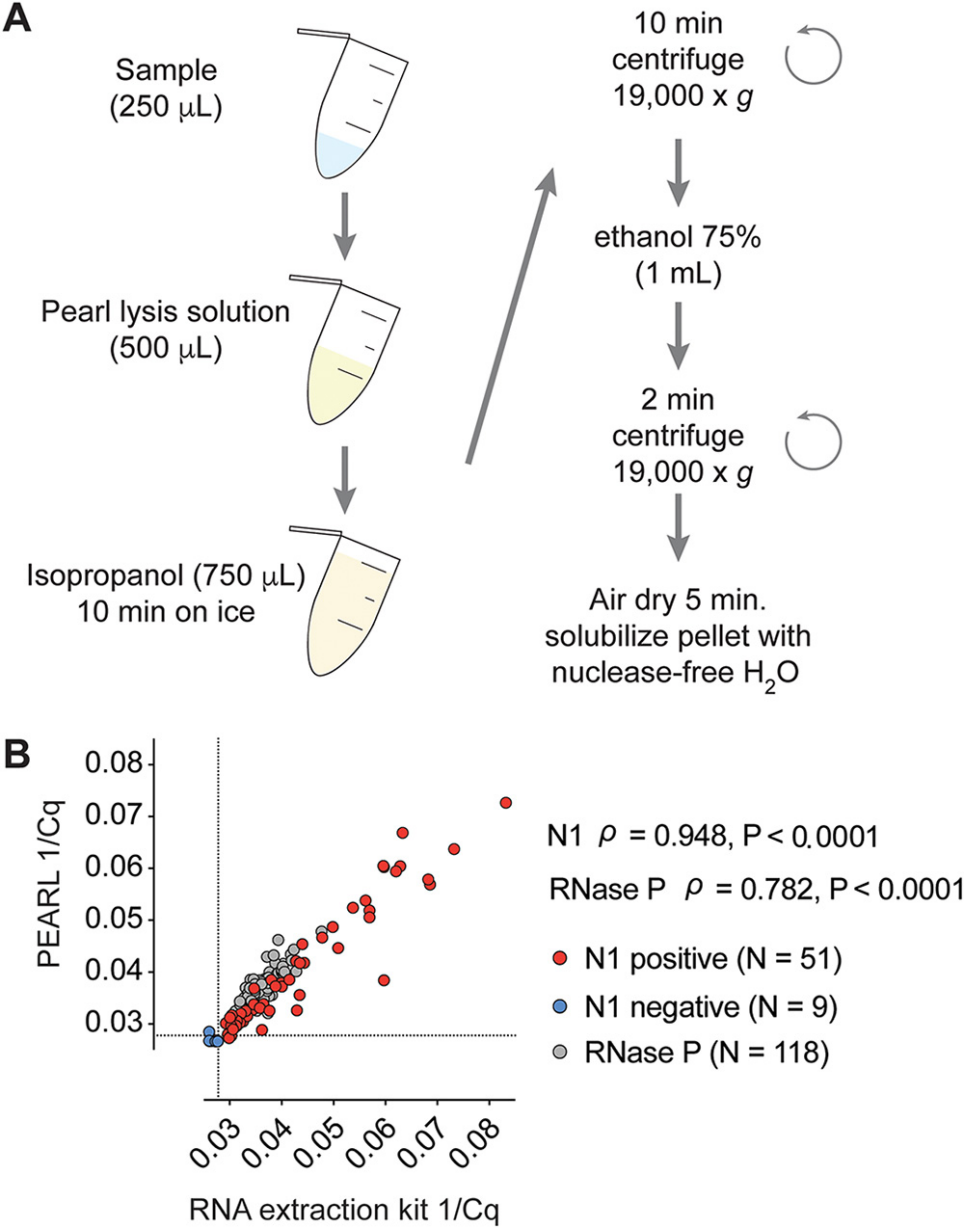
(A) PEARL workflow. (B) Comparative RT-qPCR analysis of the levels of SARS-CoV-2 nucleocapsid (N1) and RNase P RNA sequences in deidentified SARS-CoV-2-positive and -negative clinical specimen samples after RNA extraction using PEARL or an RNA extraction kit (QIAamp Mini Elute virus spin kit) in nasopharyngeal swab samples. Dotted lines indicate a limit of detection of 36 cycles. We obtained *C_q_* values for N1 below our limit of detection in 9 negative samples out of 67. No *C_q_* values were obtained for N1 in the remaining 58 samples. Data points correspond to the reciprocal of the *C_q_* value (1/*C_q_*), which is directly proportional to input RNA. *P* values and Spearman’s correlation coefficient (ρ) are shown.

We designed PEARL to provide a low-cost, column-free approach for the isolation of nucleic acids and proteins. PEARL uses common laboratory reagents (see Table S1 in the supplemental material) to recover target analytes by precipitation. Briefly, a sample is mixed with PEARL lysis solution, which disrupts cell membranes and viral envelopes, liberating DNA, RNA, and proteins. These analytes are subsequently recovered by alcohol-based precipitation and centrifugation (Fig. 1A). The PEARL lysis solution (see Materials and Methods) contains the nonionic detergent octylphenoxypolyethoxyethanol (IGEPAL-CA-630), which solubilizes biological membranes (13). The solution is neutrally buffered with HEPES (pH 7.2) to preserve macromolecule integrity and is supplemented with Tris(2-carboxyethyl)phosphine hydrochloride (TCEP), a reducing agent that protects RNA from nucleases (14). Glycerol, a low-molecular-weight crowding agent, together with sodium acetate and linear polyacrylamide (LPA), aids in precipitating DNA, RNA, and proteins (15, 16). After testing the effect of different crowding agents (glycerol and polyethylene glycol) and reducing agents (dithiothreitol and TCEP) on the detection of host and SARS-CoV-2 transcripts (Table S2), we determined an optimal concentration of 10% glycerol and 20 mM TCEP for our PEARL lysis buffer formulation. To benchmark our method, we extracted RNA from deidentified SARS-CoV-2-positive samples using PEARL or a dedicated RNA extraction kit (QIAamp Mini Elute virus spin kit; Qiagen). Next, we used the isolated RNA to examine the levels of the SARS-CoV-2 nucleoprotein (N) gene as well as the host RNase P mRNA in the samples using the 1-step reverse transcription qPCR reference test for COVID-19 recommended by the U.S. Centers for Disease Control and Prevention (CDC) (TaqMan RNA-to-Ct 1-Step kit; Thermo Fisher). In these experiments, we detected the SARS-CoV-2 N1 site using the qPCR primers and probes recommended by the CDC.

To maximize SARS-CoV-2 detection sensitivity, we tested various ratios of sample to PEARL lysis solution. We observed that 250 μl of initial swab sample input and 500 μl of PEARL lysis solution resulted in the lowest RT-qPCR *C_q_* values (Fig. S1). Next, we compared the RT-qPCR detection sensitivity obtained with PEARL extracts to that of RNA purified using a commercial kit. We found strong concordance between extraction methods using deidentified clinical specimen samples (*P* < 0.0001 for N1 SARS-CoV-2 and RNase P) (Fig. 1B). However, PEARL required a modest increase in initial sample input (1.25-fold) to achieve sensitivity similar to that of the commercial RNA extraction kit we used (Fig. 1B; note that the sample input for PEARL was either 175 μl or 250 μl, while the sample input for the commercial kit we used was 200 μl or 140 μl). Together, these results indicate that PEARL can be used as an alternative to commercial RNA extraction kits without substantial loss in sensitivity.

We reasoned that because DNA and protein coprecipitate with RNA upon addition of isopropanol during extraction (15), PEARL could be used to streamline the retrieval of RNA, DNA, and proteins from other viruses. To test whether PEARL can be used to detect different types of viruses, we used cells infected with Kaposi’s sarcoma-associated herpesvirus (KSHV), which contains a DNA genome, or with Zika virus (ZIKV), a flavivirus that contains an RNA genome and no DNA replication intermediates in its life cycle (17). In these experiments, we used iSLK-219 cells, which are latently infected with a GFP-expressing recombinant KSHV (12), or HeLa cells infected with the PRVABC59 strain of ZIKV, which was isolated in Puerto Rico in 2015 (18), at a multiplicity of infection (MOI) of 1. We collected 100,000 cells, which corresponds to the estimated cellular yield of a typical buccal swab (19), and prepared 10-fold dilutions to determine the detection limit for RNA, DNA, and protein. Next, we prepared PEARL extracts and probed for viral and host nucleic acids and proteins using qPCR- and immunodetection-based assays, respectively. To ensure the specificity of RNA or DNA detection, we treated the PEARL extracts with DNase I (to detect RNA) or RNase A (to detect DNA). For protein immunodetection, we treated the PEARL extracts with RNase and DNase before SDS-PAGE and Western blotting to ensure undisturbed migration of the proteins during electrophoresis or left them untreated for dot blot detection. Quality control analyses on PEARL extracts verified the integrity of extracted RNA, DNA, and proteins (Fig. S2).

**FIG 2:**
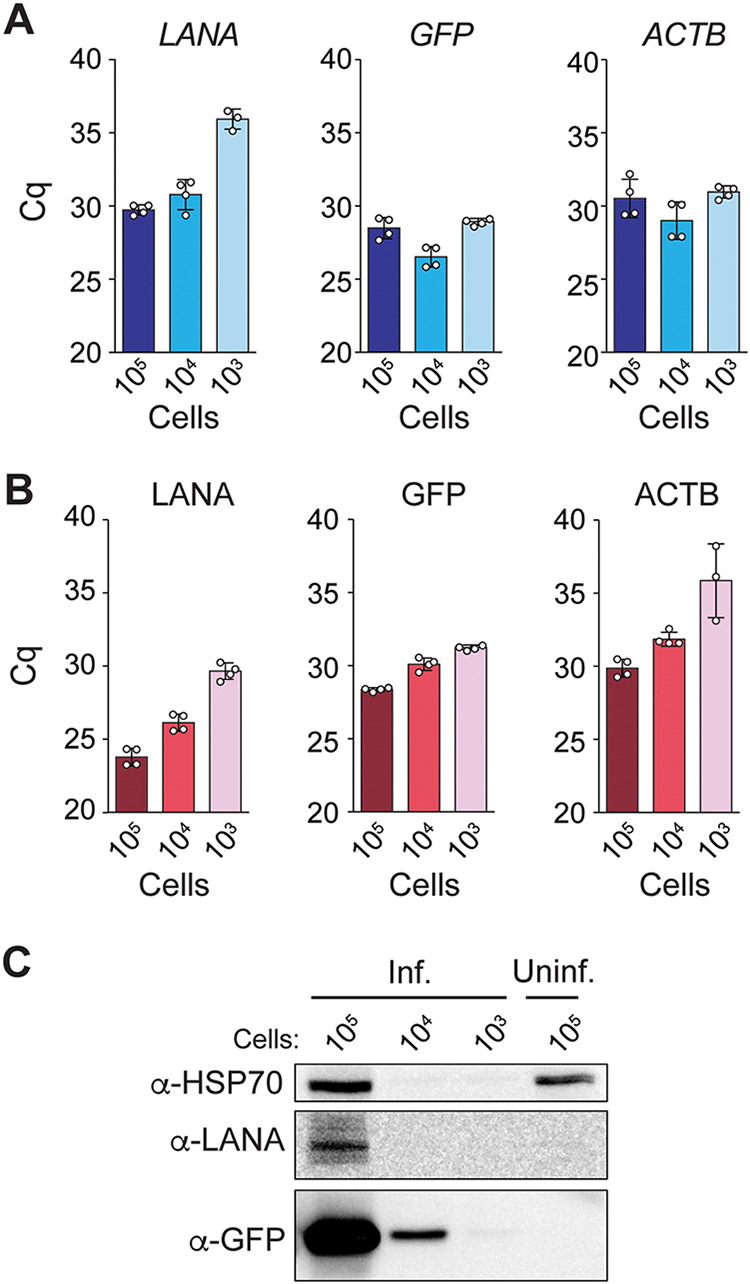
(A) RT-qPCR analysis of the levels of KSHV (LANA and GFP) and host β-actin (ACTB) mRNAs and (B) their corresponding genomic sequences. (C) Western blot analysis of the expression of KSHV (LANA and GFP) and host (HSP70) proteins. Inf., infected; Uninf., uninfected.

**FIG 3:**
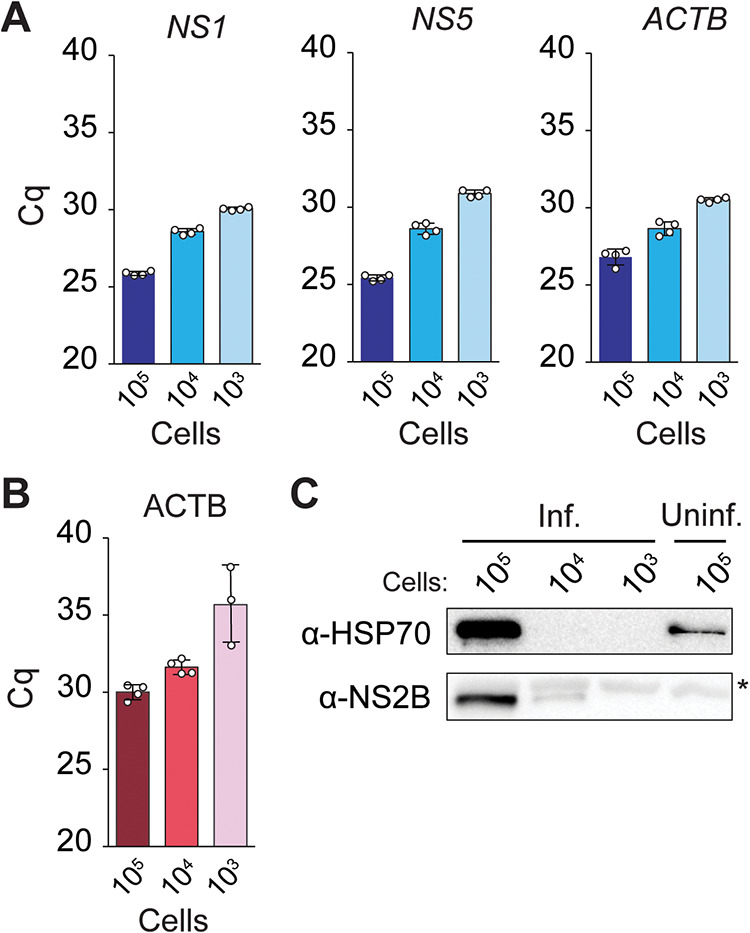
(A) RT-qPCR analysis of the levels of ZIKV nonstructural proteins NS1 and NS5 and host β-actin (ACTB) mRNAs. (B) qPCR analysis of the expression host ACTB genomic DNA sequences in ZIKV-infected samples. (C) Western blot analysis of expression of ZIKV (NS2B) and host (HSP70) proteins. *, nonspecific band.

To detect host and viral transcripts, we synthesized first-strand cDNA from the DNase I-treated samples and used it for qPCR detection of the host β-actin mRNA (*ACTB*) and viral transcripts. These viral mRNAs included those for the KSHV latency-associated nuclear antigen (LANA) and KHSV-encoded GFP (Fig. 2A), as well as ZIKV RNA regions encoding the nonstructural proteins NS1 and NS5 (Fig. 3A). In these experiments, we detected viral transcripts in PEARL extracts obtained from as few as 1,000 infected cells, and we did not observe significant differences in sensitivity between the detection of KSHV and ZIKV transcripts. Thus, PEARL can facilitate the detection of mRNAs from DNA and RNA viruses.

To detect host and KSHV genomic sequences, we used PEARL extracts treated with RNase A. Our target sequences for DNA detection corresponded to the genes for the aforementioned host and viral transcripts (Fig. 2A and 3A). In agreement with our observations for KSHV transcripts, we detected the viral genome in as few as 1,000 latently infected cells (Fig. 2B). We also detected the host DNA β-actin locus in all samples, regardless of the infection status (Fig. 2B and 3B). In these experiments, we used the same pair of PCR primers for detection of the β-actin mRNA and genomic DNA sequences, thus eliminating variability that could arise from dissimilar amplification efficiencies of different primer pairs. The primers target sequences in different β-actin exons (Fig. S3A), distinguishing mRNA amplicons from genomic DNA amplicons by molecular size. As a control, and to corroborate that the amplification products in Fig. 2B and 3B were not derived from contaminant RNA templates, we used PCR primers that amplify the nontranscribed promoter region of the host gene HSPA5 (Fig. S3B). As expected, we detected an amplification product only in the PEARL extracts treated with RNase A, not in those treated with DNase I (Fig. S3D and E), verifying the specificity of the amplification reaction.

An additional benefit of PEARL over column-based commercially available RNA extraction methods is that it allows the recovery of proteins in addition to nucleic acids. To confirm the presence of host and viral proteins in PEARL extracts, we carried out Western blot and dot blot assays using antibodies against the ubiquitous host chaperone HSP70 and the viral proteins KHSV LANA, KSHV-encoded GFP, and ZIKV NS2B. In these experiments, we detected the host chaperone HSP70 in PEARL extracts obtained from 100,000 cells (HeLa and iSLK-219) by Western blotting (Fig. 2C and 3C) and in as few as 12,500 cells (HeLa and iSLK-219) by dot blotting (Fig. S4A and B). Detection of KSHV-encoded GFP was achievable with approximately 1,000 iSLK-219 cells (Fig. 2C; Fig. S4B). Detection of KSHV-LANA was significantly less sensitive by Western blotting than by dot blotting, requiring 100,000 and 1,250 iSLK-219 cells, respectively (Fig. 2C; Fig. S4B). Taken together, our results indicate that PEARL can be used as a reliable and efficient method to extract host and virus nucleic acids and proteins from a wide range of viral infections.

While we designed PEARL to be accessible, it still uses a high-speed centrifuge, which is expensive, requires AC power to operate, and is typically restricted to professional laboratories. We reasoned that we could make PEARL field deployable by using a hand-powered centrifugation device. Inspired by the work of Byagathvalli et al. and of the Prakash lab, who pioneered these types of devices (20–22), we modified a freely available design for a hand-powered centrifuge actuated by supercoiling of a string (https://www.thingiverse.com/thing:1946291). We engineered a safety lid and chamfered all edges to avoid abrasion of the string, and we increased the distance between the pull points to augment torque around the rotational axis (Fig. S5A). We 3D printed our device with thermoplastic polyester, measured its angular velocity using a laser tachometer, and found that we could achieve centrifugal forces of approximately 3,900 × *g* (Fig. S5B). To test whether our hand-powered centrifuge could replace a benchtop centrifuge in PEARL, we compared the RNA and protein extraction efficiency achieved with our device and with a benchtop centrifuge set at 19,000 × *g*. Despite the substantial difference in centrifugal force, we found that viral RNA and proteins can be easily detected using PEARL extracts prepared with our hand-powered centrifuge (Fig. S5C and D). Thus, this device can enable the deployment of PEARL in the field without a significant loss in detection sensitivity.

## DISCUSSION

The primary tool to combat the COVID-19 pandemic is widespread and accessible testing to monitor SARS-CoV-2 prevalence and spread, which informs deployment of containment and mitigation measures (23). Globally scaled testing remains an unmet public health need, as attempts to meet this demand have resulted in shortages of the reagents and supplies necessary for sample processing, RNA extraction and SARS-CoV-2 detection. Here, we present data to support PEARL as a cost-effective, simple, and less-toxic alternative for the isolation of RNA, DNA, and proteins. Our results indicate that PEARL enables the detection of SARS-CoV-2 transcripts in COVID-19-positive swab samples with sensitivity comparable to that afforded by commercially available RNA extraction kits. This outcome highlights the validity of using PEARL as a viable alternative to facilitate the detection of SARS-CoV-2 in respiratory samples.

Our data also show that PEARL extracts can be used to efficiently detect host and viral transcripts, genomic DNA, and proteins regardless of the nature of the infection—PEARL was equally useful in detecting DNA and RNA viruses with different tropism. Coupling PEARL to different downstream analyses for detection of nucleic acids and proteins can provide a powerful tool for detection of diverse viruses. Moreover, because RNA, DNA, and proteins are extracted at once, PEARL reduces sample handling time, allowing streamlined diagnostic procedures. Thus, it may enable both nucleic acid- and antigen-based SARS-CoV-2 testing. PEARL’s minimal handling requirements also make it scalable, which is desirable for high-volume testing operations, as is needed for SARS-CoV-2 testing.

It is possible that the collection medium used to store samples before processing may influence the performance of PEARL. For example, the viral transport medium recommended by the CDC to store and inactivate samples for SARS-CoV-2 testing (2% fetal bovine serum, 100 μg/ml gentamicin, 0.5 μg/ml amphotericin B, and various salts) (24) has components that could coprecipitate with target analytes. Isopropanol is less polar than ethanol, and therefore, it has a higher propensity to precipitate salts and antibiotics (25). In our experiments, coprecipitation of salts and antibiotics does not appear to compromise downstream RT-qPCR or immunodetection assays. Concerns regarding the inhibition of downstream detection assays could be addressed by using ethanol instead of isopropanol.

It is also possible that PEARL may introduce extraction bias, as short RNAs, including tRNAs, snoRNAs, and microRNAs (miRNAs), are more difficult to precipitate than longer RNA and DNA molecules (25). Though we have not directly tested whether small RNAs are underrepresented in PEARL extracts, we designed PEARL to enhance the precipitation of all RNAs by using linear polyacrylamide as a carrier (16). Additionally, longer centrifugation and higher centrifugation speeds can be used to enhance small-RNA recovery, if needed. Further improvements may be required to implement PEARL as mainstream nucleic acid and protein isolation tool for detection of viruses obtained from sources different from those described here, as the sample type may dictate overall performance. Future work outside the scope of this study will be required to address whether this is the case.

Finally, since PEARL uses common reagents and does not require expensive equipment or highly trained personnel, it can provide an accessible alternative for streamlining diagnostics in geographic areas that lack access to capital, specialized reagents, and professional laboratories. Moreover, PEARL is field deployable, given that a hand-powered centrifugation device can be used. In view of these considerations, coupling PEARL to our recently developed CRISPR-based protocol for detection of SARS-CoV-2 genetic material called CREST (Cas13-based, rugged, equitable, scalable testing) (26) could allow efficient, affordable, widespread testing, lowering the barrier of “luxury testing” in many regions of the world.

## Supplementary Material

Supplemental file 1
