# Supplementary material for: A Fast and Accessible Method for the Isolation of RNA, DNA, and Protein To Facilitate the Detection of SARS-CoV-2
Source: J Clin Microbiol. 2021 Mar 19;59(4):e02403-20. doi: 10.1128/JCM.02403-20 (PMC8092744; doi:10.1128/JCM.02403-20)
Supplement: Supplemental file 1 [file JCM.02403-20-s0001.pdf]

JCM02403-20

Supplemental Figures and Tables

Reagent	Cost (USD)	USD/gram/mL	For 500µL	Cost/reaction
K-Hepes	$728/ 1 Kg	$0.728	0.002383 g	0.00173482
NaOAc	$659/2000 g	$0.3295	0.0150075 g	0.00494497
Igepal CA-630	$98.2/500 mL	$0.1964	0.0025 mL	0.000491
Glycerol	$907/4000 mL	$0.22675	0.2 mL	0.04535
TCEP	$178/10 g	$17.8	0.0028665 g	0.0510237
Polyacrylamide	$173/250 g	$0.692	0.000025 g	0.0000173
Isopropanol	$100.3/1000 mL	$0.1003	$0.075	$0.075
Total				$0.17851

Supplemental Table 1. Cost breakdown of PEARL reagents. Note that the cost of one PEARL extraction is approximately 20 times lower than column-based RNA extraction.

Buffer	GAPDH	7SL	N1
CL+Glycerol	36.93	28.95	33.55
CL+Glycerol+DTT	36.69	28.00	30.82
CL+Glycerol+TCEP	35.62	27.78	30.32
CL+PEG	34.73	29.67	34.06
CL+PEG+DTT	35.15	28.97	31.84
CL+TCEP	35.13	28.39	32.38

Supplemental Table 2. Effect of different PEARL lysis components in the sensitivity of RT-qPCR-based detection of host (*GAPDH* and 7SL RNA) and a synthetic, *in vitro* transcribed, SARS-CoV-2 N1 transcript. Cq values are indicated for each transcript. Core lysis solution (CL): 450 mM NaOAc, 50 µg/mL linear polyacrylamide, 0.5% IGEPAL CA-630, 20 mM HEPES-KOH pH = 7.2. Additive concentrations as follows: 10% glycerol, 2 mM DTT, 20 mM TCEP and 10% polyethylene glycol 8000 (PEG).

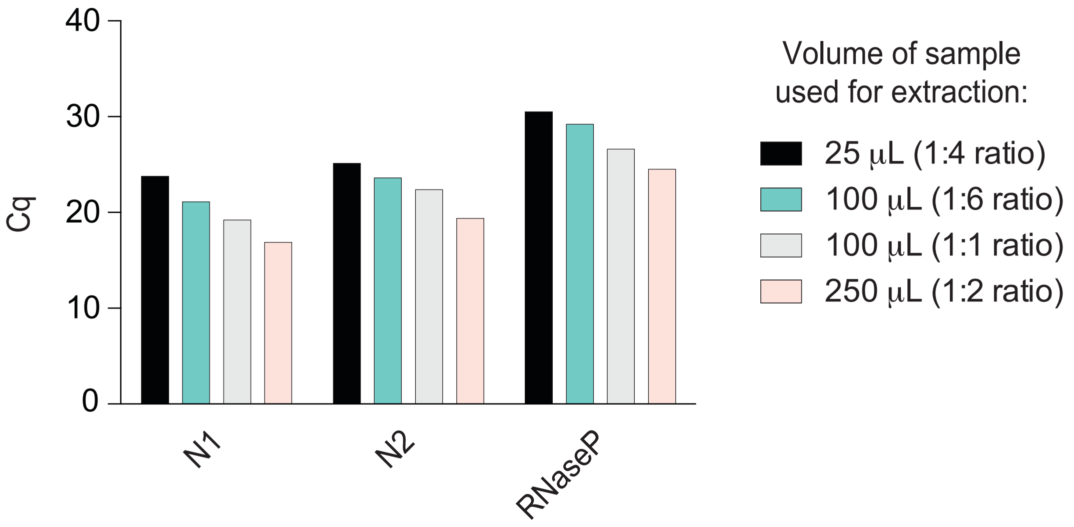

Supplemental Figure S1. Threshold values obtained by RT-qPCR for SARS-CoV-2 N1 and N2 viral transcripts, and the RNase P host mRNA, obtained using PEARL extracts. Different volumes of swab samples with the indicated sample to PEARL-lysis-buffer ratios were used to determine the conditions that maximize readout.

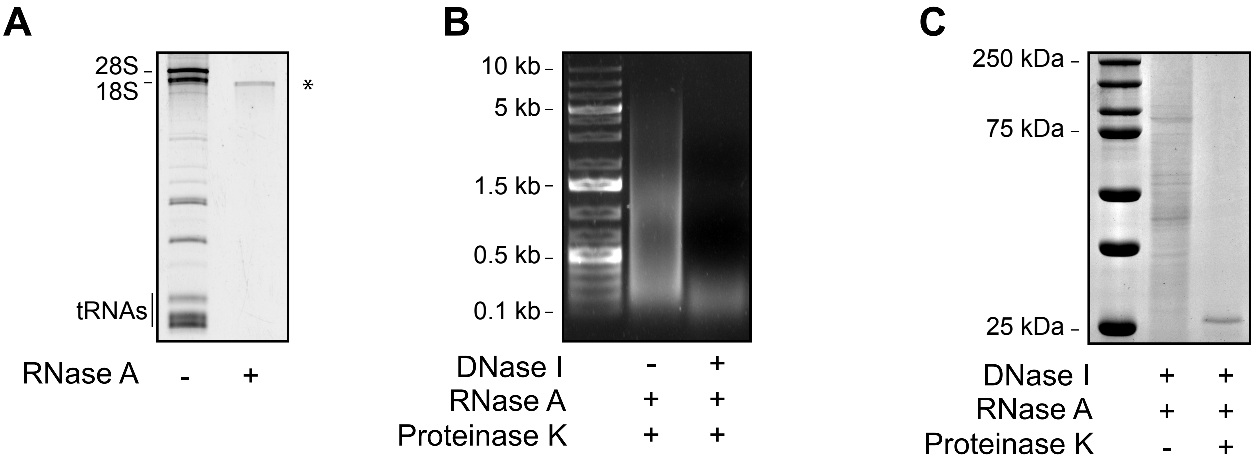

Supplemental Figure S2. Assessment of the integrity of PEARL-extracted analytes. RNA, DNA, and proteins were extracted from 1×10^6 HeLa cell using PEARL to their integrity was assessed by electrophoresis analyses. A) Total RNA isolated from PEARL extracts using TRIzol reagent. The RNA was incubated with RNase A for 15 min at 37 °C or left intact, separated on a urea-PAGE gel, and stained with SYBR Safe. *partial digestion of rRNAs in the sample. Major RNA species are indicated. B) PEARL extracted total DNA after RNA and protein digestion. Extracts were treated with DNase I or left intact as indicated, and separated on an agarose gel stained with SYBR Safe. C) PEARL protein extracts separated by SDS-PAGE and stained with Coomassie blue after nuclease digestion.

A

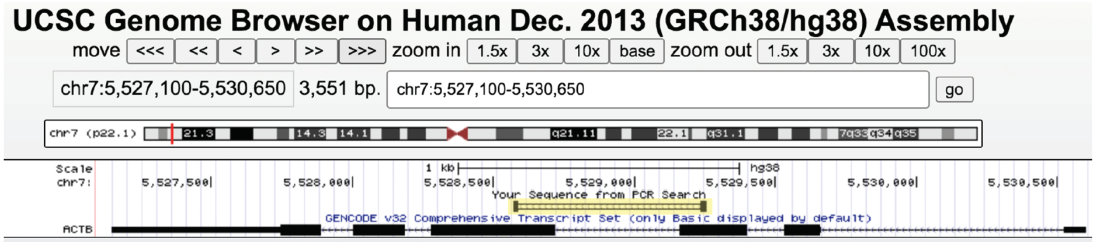

B

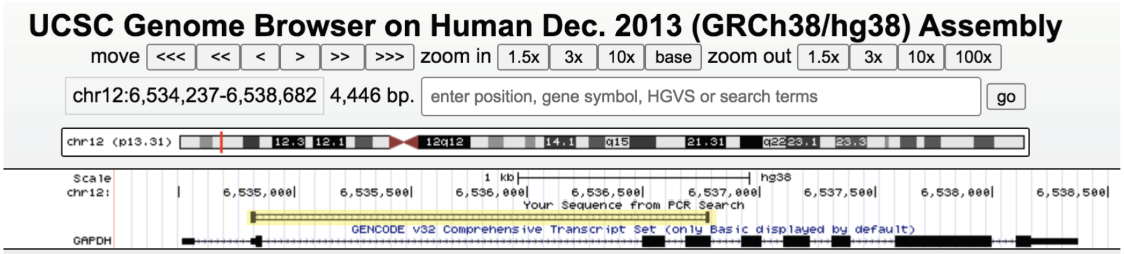

C

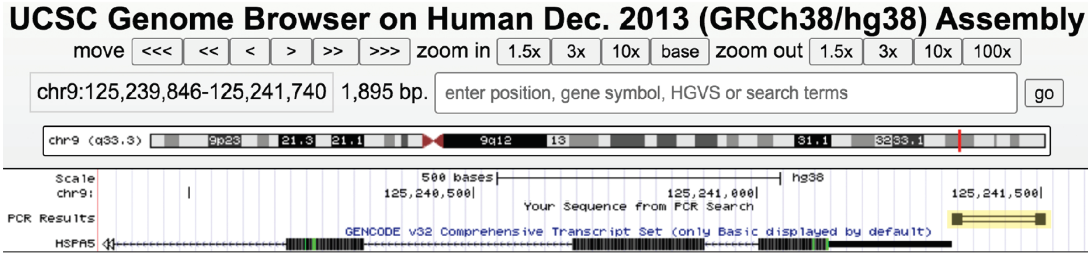

D

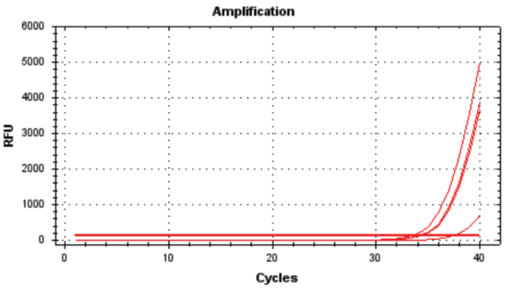

E

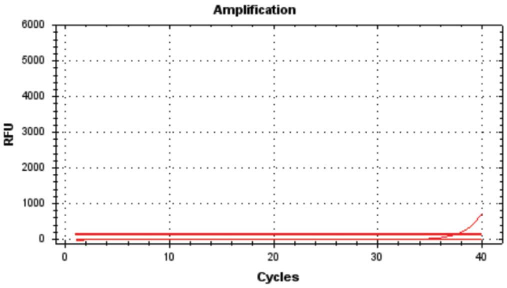

Supplemental Figure S3. Genomic locations of primer binding sites (black blocks flanking the sequences highlighted in yellow) targeting A) exons 3 and 4 in the ACTB locus, B) exons 2 and 4 in the GAPDH locus, and C) the HSPA5 proximal promoter region. Tracks were obtained by *in silico* PCR and were mapped onto the latest assembly of the human genome in the UCSC genome browser (https://genome.ucsc.edu/index.html). D) and E) Amplification curves for the HSPA5 promoter region using the PCR primers shown in B in using PEARL extracts treated with (C) RNase A or (D) DNase I.

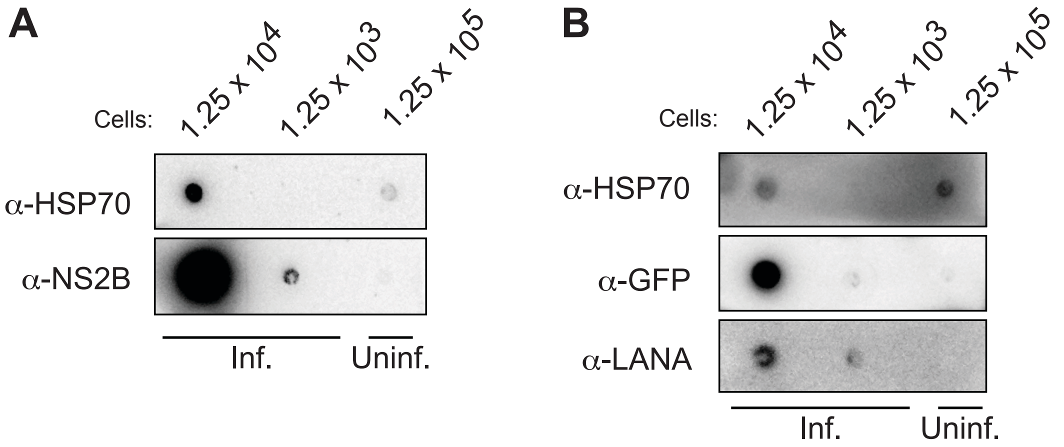

Supplemental Figure S4. A) Dot blot immunodetection of ZIKV (NS2B) and host (HSP70) proteins. B) Dot blot immunodetection of KSHV (GFP, LANA) and host (HSP70) proteins. Inf., infected; Uninf., uninfected.

A

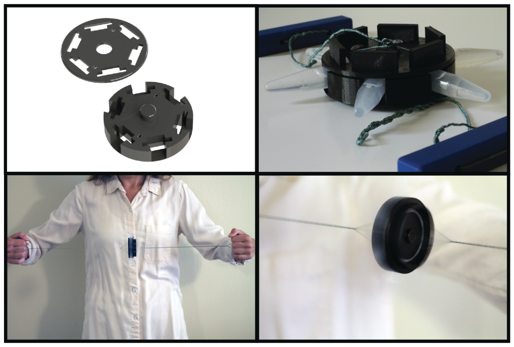

B

Pull #	RPM (Max)	RCF (Max)
1	7240	3809
2	7324	3898
3	6915	3475
4	7812	4435
5	7547	4139
6	6432	3006
7	6665	3228
8	7836	4462
9	6846	3406
10	8532	5292

Handfuge maximum speed. The handfuge rotor was populated with six tubes, each containing 1.5 ml of PBS. The centrifuge was actuated ten times and maximum speed was determined with a laser tachometer.

C

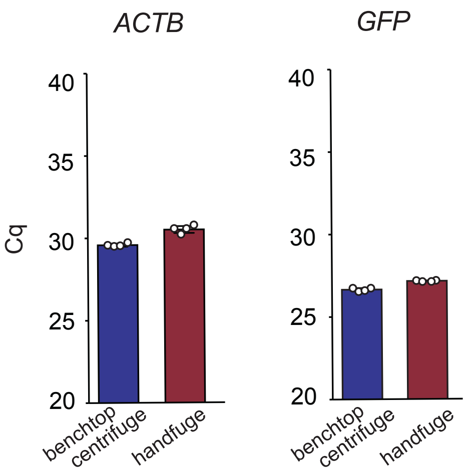

D

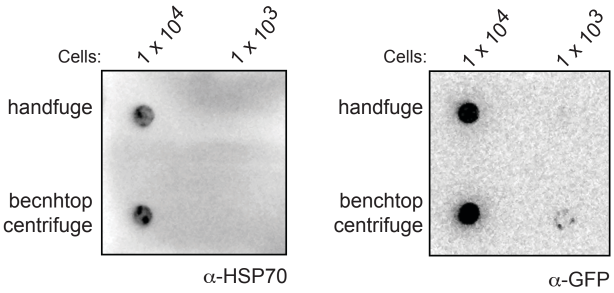

Supplemental Figure S5. A) Top left: 3D render of our hand-powered centrifuge. CAD files can be found here: https://3dprint.nih.gov/discover/3dpx-014683. Top right: Assembled hand-powered centrifuge with its rotor loaded and the driving strings supercoiled. Bottom left: Actuation of the hand-powered centrifuge by string supercoiling. Bottom right: Close-up image of the spinning rotor during actuation. B) Laser tachometer data averaged from 10 actuation events of the hand-powered centrifuge. C) Cq values obtained by probing for host and KSHV mRNAs using RT-qPCR using PEARL extracts prepared with either a benchtop laboratory centrifuge or our hand-powered centrifuge, “Handfuge”. D) Dot blots obtained by probing for host and KSHV proteins in PEARL extracts prepared as in C.

List of oligonucleotides used for qPCR in this study

Target	Organism	Forward Oligonucleotide	Reverse Oligonucleotide
ACTB	*Homo sapiens*	TTCTACAATGAGCTGCGTGTG<br>exon 3; chr7:5529236-5529256	AGGGCATACCCCTCGTAGAT<br>exon 4; chr7: 5528571-5528590
HSPA5 (promoter)	*Homo sapiens*	GCGGAGCAGTGACGTTTATT<br>chr9:125241491-125241510	ACCTCACCGTCGCCTACTC<br>chr9:125241345-125241363
GAPDH	*Homo sapiens*	AGCCACATCGCTCAGACAC<br>exon 2; chr12:6534813-6534831	TGGAAGATGGTGATGGGATT<br>exon 3; chr12:6536768-6536787
NS1	Zika Virus PRABC59	ATAACAGCTTTGTCGTGGATG	TAACCTTGAGCCAGACACTAG
NS5	Zika Virus PRABC59	GACTGGGTTCCAACTGGGAG	CCACACTCTGTTCCACACCA
GFP	KSHV	GAGCGCACCATCTTCTTCAAG	GGCGGATCTTGAAGTTCAC
LANA	KSHV	CCTCCATCCCATCCTGTGTC	GGACGCATAGGTGTTGAAGAG
